# Junior Editor of the Review

**Published:** 1858-07

**Authors:** 


JUNIOR EDITOR OF THE REVIEW.—The Senior Editor takes pleasure in announcing that his colleague, Dr. T. G. Richardson, has been appointed to the Chair of Anatomy in the Medical Department of the University of Louisiana, vacant by the resignation of Dr. Nott, who has resumed his practice at Mobile. Dr. Richardson, during his brief sojourn in this city, discharged the duties of his Chair in the Pennsylvania Medical College with signal ability, and made many warm personal friends, both in and out of the profession, who deeply regret his departure. They are consoled, however, by the fact that the field upon which he is about to enter is one of great honor and usefulness, affording him ample scope for the exercise of his talents as a teacher, writer, and practitioner.

The removal of Professor Richardson will in no wise interfere with the interests of the Review; on the contrary, it is believed that it will augment both its usefulness and its influence. Connected as he now will be, by virtue of his Chair in the University of Louisiana, with the Charity Hospital at New Orleans, one of the greatest eleemosynary institutions of the kind in America, it will be a part of his business to furnish for the Journal, from time to time, extended reports of what he and others may see and do in that establishment. In this manner, and by his influence in obtaining new collaborators, the Journal, while it will be made the vehicle of contributions full of interest to Southern practitioners, because illustrative of Southern diseases and Southern modes of practice, will assume still more of that broad, national character to which it originally aspired, and which, through the ability of its collaborators, including some of the best talent and erudition of the country, and the distinguished liberality of the enterprising publishers, it is no vain-boasting to declare, it has already attained. The arrangement of the matter, the correction of the press, and the editorial correspondence will hereafter be performed by the Associate Editor, S. W. Gross, M.D.

